# Navigator-gated 3D cine DENSE: development and initial evaluation

**DOI:** 10.1186/1532-429X-11-S1-O94

**Published:** 2009-01-28

**Authors:** Xiaodong Zhong, Bruce S Spottiswoode, Craig H Meyer, Christopher M Kramer, Frederick H Epstein

**Affiliations:** 1 University of Virginia, Charlottesville, VA USA; 2 University of Stellenbosch, Tygerberg, South Africa

**Keywords:** Longitudinal Strain, Entire Left Ventricle, Rapid Data Acquisition, Navigator Gating, Routine Clinical Imaging

## Introduction

Ideally, imaging of cardiac function should cover the entire heart and completely quantify myocardial deformation in three dimensions.

## Purpose

To (a) develop a free-breathing navigator-gated 3D cine DENSE (Displacement-encoding with Stimulated Echoes) pulse sequence to acquire such data and (b) implement post-processing methods to quantify 3D myocardial strain throughout the left ventricle (LV).

## Methods

An ECG-gated segmented 3D spiral cine DENSE pulse sequence with navigator gating and online image reconstruction was implemented on a 1.5 T MRI scanner (Siemens Avanto, Erlangen, Germany). A 3D stack of spirals *k*-space trajectory was employed for rapid data acquisition. Three-point phase cycling was used for artifact suppression, a balanced four-point method was used for optimal displacement encoding, and field map acquisition and online spiral deblurring were employed. A navigator echo was placed at the end of the cardiac cycle, so as not to interfere with imaging of the onset of myocardial contraction. The navigator echo was used to accept or reject the DENSE data acquired in the subsequent heart beat. Five normal volunteers provided informed consent and were studied in accordance with protocols approved by our institutional review board. Imaging parameters included voxel size = 2.8 × 2.8 × 5.0 mm^3^, flip angle = 20°, TR = 16 ms, TE = 1.3 ms, number of spiral interleaves = 6, temporal resolution = 32 ms, and cardiac phases = 22. A double-oblique 3D volume was aligned with the short and long axes of the LV. Fourteen *k*-space partitions were acquired and then zero-padded to reconstruct 28 slices. A 3 mm navigator acceptance window was placed at the end-expiration position. For displacement and strain analysis, images were exported to a PC and manually segmented. Tissue tracking and strain analysis were performed using 3D extensions of 2D methods that were described previously. To validate the 3D measurements, separate 2D cine DENSE MRI was also performed in multiple short- and long-axis planes. Non-navigator-gated 3D data were also acquired in some volunteers.

## Results

**Figure 1:**
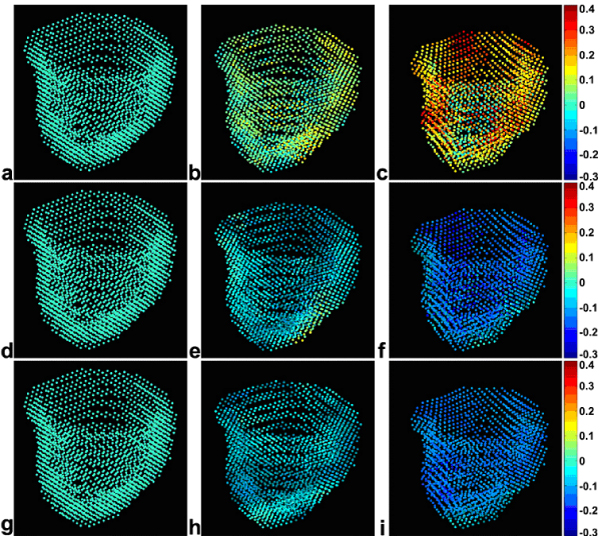
**Radial (a-c), circumferential (d-f), and longitudinal (g-i) strains computed throughout the LV from 3D cine DENSE MRI of a normal volunteer**. Data are shown at end diastole (a, d, g), mid systole (b, e, h), and end systole (c, f, i). Three of 22 cardiac phases are displayed. Individual dots represent displacement, while color represents strain.

Using a 3 mm navigator acceptance window, the acceptance rate was 48.0 ± 15.7% and the total scan time was 20.5 ± 5.7 minutes. High-quality data were acquired from all volunteers, and comparisons with non-navigator-gated free breathing scans clearly demonstrated the reduction of respiratory artifacts provided by navigator gating. Typical 3D strain data from one subject are shown in Fig. 1, where the development of radial (a-c), circumferential (d-f), and longitudinal strain (g-i) from end diastole (a, d, g) through mid systole (b, e, h) to end systole (c, f, i) are displayed for the entire LV. Strain values and strain-time curves were consistent with previous data from myocardial tagging and DENSE studies of normal volunteers. For the comparison with 2D cine DENSE, linear regression showed that radial, circumferential, and longitudinal strains from 3D cine DENSE correlated well with those from 2D cine DENSE, with a slope of 0.974 and R = 0.647 for radial strain, a slope of 0.945 and R = 0.902 for circumferential strain, and a slope of 0.888 and R = 0.772 for longitudinal strain. Bland-Altman analysis also demonstrated good agreement between the 2D and 3D cine DENSE methods for all 3 strains.

## Conclusion

A free-breathing navigator-gated 3D cine DENSE pulse sequence was developed that provides high spatial and temporal resolutions, coverage of the entire LV, and measurement of 3D strain with a scan time of approximately 20 minutes. In normal volunteers, the resulting strain data show good agreement with those from 2D cine DENSE. With additional development aimed at further shortening the scan time and automating image analysis, these methods may enable routine clinical imaging that completely quantifies contractile function throughout the LV in patients with contractile dysfunction.

